# Superiority of Droplet Digital PCR Over Real-Time Quantitative PCR for *JAK2* *V617F* Allele Mutational Burden Assessment in Myeloproliferative Neoplasms: A Retrospective Study

**DOI:** 10.3390/diagnostics10030143

**Published:** 2020-03-05

**Authors:** Francesco La Rocca, Vitina Grieco, Vitalba Ruggieri, Emanuela Zifarone, Oreste Villani, Pietro Zoppoli, Sabino Russi, Simona Laurino, Geppino Falco, Giovanni Calice, Anna Marinaccio, Maria Iole Natalicchio, Francesco Albano, Pellegrino Musto

**Affiliations:** 1 Laboratory of Clinical Research and Advanced Diagnostics, IRCCS-CROB, Referral Cancer Center of Basilicata, 85028 Rionero in Vulture (Pz), Italy; francesco.larocca@crob.it (F.L.R.); vitina.grieco@crob.it (V.G.); 2 Laboratory of Preclinical and Translational Research, IRCCS-CROB, Referral Cancer Center of Basilicata (CROB); 85028 Rionero in Vulture (Pz), Italy; pietro.zoppoli@crob.it (P.Z.); sabino.russi@crob.it (S.R.); simona.laurino@crob.it (S.L.); giovanni.calice@crob.it (G.C.); 3 Trial Office, IRCCS-CROB, Referral Cancer Center of Basilicata, 85028 Rionero in Vulture (Pz), Italy; emanuela.zifarone@crob.it; 4 Hematology and Stem Cell Transplantation Unit, IRCCS-CROB, Referral Cancer Center of Basilicata, 85028 Rionero in Vulture (Pz), Italy; oreste.villani@crob.it; 5 Department of Biology, University of Naples Federico II, 80138 Naples, Italy; geppino.falco@unina.it; 6 Biogem, Istituto di Biologia e Genetica Molecolare, Via Camporeale, 83031 Ariano Irpino (AV), Italy; 7 Section of Clinic Pathology, OO.RR., 71122 Foggia, Italy; marinaccioanna@gmail.com (A.M.); iole.nat@tiscali.it (M.I.N.); 8 Unit of Hematology and Stem Cell Transplantation, AOU Policlinico Consorziale “Giovanni XXIII”, “Aldo Moro” University, 70124 Bari, Italy; francesco.albano@uniba.it (F.A.); pellegrino.musto@uniba.it (P.M.)

**Keywords:** myeloproliferative neoplasms, qPCR, droplet digital PCR, *JAK2*

## Abstract

*JAK2V617F* mutational status is an essential diagnostic index in myeloproliferative neoplasms (MPNs). Although widely used for detection of *JAK2 V617F* mutation in peripheral blood (PB), sensitive real-time quantitative PCR (qPCR) presents some methodological limitations. Recently, emerging alternative technologies, like digital droplet PCR (ddPCR), have been reported to overcome some of qPCR’s technical drawbacks. The purpose of this study was to compare the diagnostic utility of ddPCR to qPCR for *JAK2 V617F* detection and quantification in samples from MPNs patients. Sensitivity and specificity of qPCR and ddPCR in the detection of the mutation were assessed by using a calibrator panel of mutated DNA on 195 *JAK2* positive MPN samples. Based on our results, ddPCR proved to be a suitable, precise, and sensitive method for detection and quantification of the *JAK2 V617F* mutation.

## 1. Introduction

Detection of the *JAK2 V617F* variant in patients with Philadelphia-negative myeloproliferative neoplasms (MPNs), including polycythemia vera (PV), essential thrombocythemia (ET), and primary myelofibrosis (PMF) has changed the MPN molecular diagnostics routine over recent years [1,2]. The mutation occurs in more than 90% of PV cases and in about 50–60% of those with ET or PMF. Interestingly, several studies showed that different MPNs phenotypes correlate with the mutant allelic burden [3,4]. Patients with ET, for instance, have the lowest allele burden (<50%) when compared to post-PV myelofibrosis (>90%) [5,6]. In fact, the presence of an allele burden greater than 50% increases the probability of an overt PV or myelofibrotic evolution [7,8,9]. In addition, a *JAK2 V617F* allele burden may have prognostic significance as well, since it correlates with clinical endpoints in MPN patients [10]. Quantitative real-time PCR (qPCR) has been widely applied to nucleic acid-based diagnostic tests, but lack of standardization and a relatively poor accuracy have hindered its usefulness in some clinical applications. Droplet digital PCR (ddPCR) is a novel PCR-based technology that has already been successfully used for sensitive and reproducible detection of different pathogenetic variants [11,12].

As ddPCR allows robust and precise quantification of nucleic acid copies without the need for any calibration curve, it appears to be more appropriate for the analysis of mutation allele burden [13,14] and, thus for a better clinical management of patients, in the era of JAK-inhibitor therapy.

Here, we reported the results of our retrospective single cohort study on 195 *JAK2* positive patients with MPNs performed by using qPCR and ddPCR for the evaluation of the *JAK2 V617F* mutation allele burden. Although other similar studies addressing this issue have been published, to the best of our knowledge, we compared, for the first time, our determinations with reference samples with standardized amounts of mutated variant allele. Using external calibrators is crucial for standardizing measurements and for assessing the commutability of reference materials in laboratory procedures. We concluded that, compared to qPCR, ddPCR is a significantly more precise, sensitive, and reproducible method for *JAK2 V617F* detection.

## 2. Materials and Methods

### 2.1. Patients’ Samples

Peripheral blood samples were collected from 195 *JAK2*-positive MPN patients at time of diagnosis and from 50 healthy controls at the Department of Hematology, IRCCS-CROB (Referral Cancer Center of Basilicata, Rionero in Vulture, Italy). The study was approved by the Ethics Committee of IRCCS-CROB (CEUR 141/2014, 18 November 2014) and all patients provided informed consent according to the Declaration of Helsinki. Clinical characteristics of patients are described in Table 1.

### 2.2. Reference Samples

A panel of seven freeze-dried genomic DNA samples with different percentages of *JAK2 V617F* mutation (0%, 0.03%, 1.00%, 10.8%, 29.6%, 89.5%, 100%) was used as calibrator panel (National Institute for Biological Standards and Control, NIBSC, WHO Reference Panel 1st International Reference Panel for Genomic *JAK2 V617F*, version 3.0).

### 2.3. DNA Extraction

Genomic DNA was extracted from peripheral blood samples. DNA was obtained out of 400 µL of whole blood in the MagNA Pure Compact Instrument (Roche Diagnostics, Mannheim, Germany). The DNA was diluted with nuclease-free water (Ambion, Austin, TX, USA) in every case at 12.5 ng/µL.

### 2.4. qPCR

Real-time PCR was carried out with the Probe Master (Roche Diagnostics). Extracted DNA, a total of 25 ng, was added to the PCR mixture containing MgCl_2_, primers and probes according to the Ipsogen *JAK2* MutaQuant (Qiagen, Hilden, Germany) recommendations. The conditions were as follows: initial denaturation of 1 cycle of 10 min at 95 °C, followed by 45 cycles of 15 s at 95 °C and 90 s at 63 °C. Each sample was analyzed in triplicate and, to monitor contamination, a negative sample and a DNase- and RNase-free sterile water control were included in each PCR run. Plasmids of known concentrations provided were used as a reference internal standard for the calculation of copy number of both wild-type and mutated standards. The concentration of target DNA was calculated by plotting the crossing point of each sample on the standard curves by using the Light Cycler software by using LightCycler 480 system (Roche).

### 2.5. ddPCR

The Bio-Rad QX200 system (Bio-Rad Laboratory, Hercules, California, USA) was used to perform ddPCR. In each ddPCR reaction, a FAM-labeled probe for the *JAK2 V617F* mutation and a HEX-labeled probe for the *JAK2 V617F* wild-type allele were used (Bio-Rad, UniqueAssayID dHsaMDV2010061). The reaction volume was carried out in 20 μL as described: 10 μL of 2X ddPCR Supermix for Probes No dUTP (Bio-Rad Laboratory), 2 μL of 20X FAM/HEX hydrolysis probes, 4 μL nuclease-free water and 4 μL of genomic DNA (25 ng). PCR was performed in a Veriti PCR thermocycler (Thermo Fisher) using the following conditions: 1 cycle at 95 °C for 10 min, followed by 40 cycles at 95 °C for 30 seconds and 55 °C for 60 s, and followed by 1 cycle at 98 °C for 10 min (ramp rate 2.2 °C/s). After PCR, the 96-well plate was loaded in the QX200 droplet reader. The droplets from each well were analyzed according to the Poisson distribution and the absolute copy number of the *JAK2 V617F* and wild-type *JAK2* alleles was calculated using the QuantaSoft analysis software (Bio-Rad Laboratory). The percentage of *JAK2 V617F* mutated alleles was calculated as copy number (mutated / (mutated + wild−type) × 100). The samples were analyzed in duplicates.

### 2.6. Statistical Analysis

The GraphPad version 6.0 software (La Jolla, CA, USA) and R were used to calculate the statistical parameters. *V617F* mutation allele burdens in clinical samples assessed by qPCR and ddPCR were compared using the Spearman rank correlation coefficient analysis. Bland–Altman analysis [15] was used to evaluate the agreement between the two PCR methods in clinical samples. Moreover, a comparison between allele burden medians measured by both methods separately for different MPNs was performed by the Mann–Whitney test. 

For the qPCR method, the limit of blank (LoB) was established by measuring false positive events from a series of 50 wild-type control samples by using the formula LoB = mean (blank) + 1.645x standard deviation (SD, blank). The limit of detection (LoD) was established for both methods by measuring the lowest genomic DNA concentration contained within the NIBSC calibrator (0.03% *JAK2 V617F*). For the qPCR, LoD was established by using the formula LoD = LoB + 1.645x SD (0.03% *JAK2 V617F*) and analyzing a series of 50 replicates of the lowest concentration sample (0.03% *JAK2 V617F*).

For the ddPCR method, LoB was calculated as the average (%) of false positive events measured in 50 wild-type control samples, in triplicate. The LoD was calculated by applying the formula: LoD = LoB + 3x SD (0.03% *JAK2 V617F*) [16].

## 3. Results

**Figure 1:**
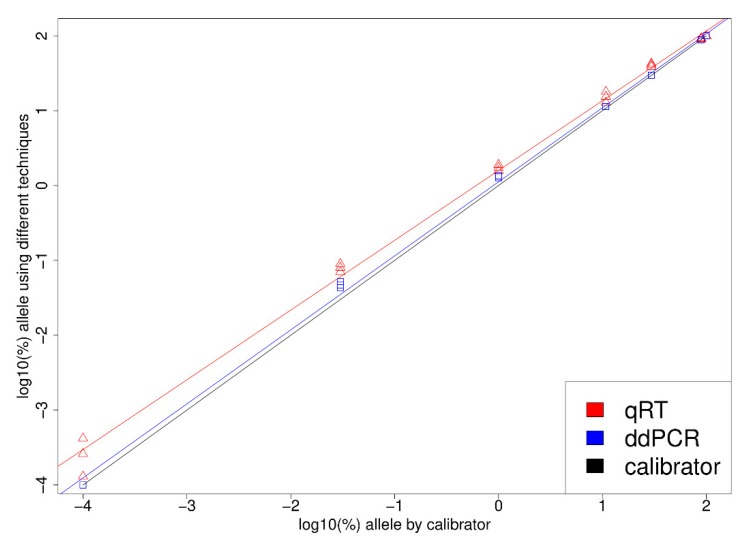
Log–log plot demonstrating the correlation of droplet digital CR (ddPCR) and qPCR *JAK2 V617F* mutation allele frequency quantification with NIBSC calibrators.

The analytical performance results of qPCR and ddPCR methods were assessed using WHO calibrators. A total of three replicates for each sample concentration were analyzed. Data from calibrators, measured by qPCR and ddPCR, showed a positive linear relationship. The R-squared measures were 0.999 and 0.995 for qPCR and ddPCR, respectively. After converting to log–log scale, linear regression showed a significant concentration-dependent bias (Figure 1).

**Figure 2:**
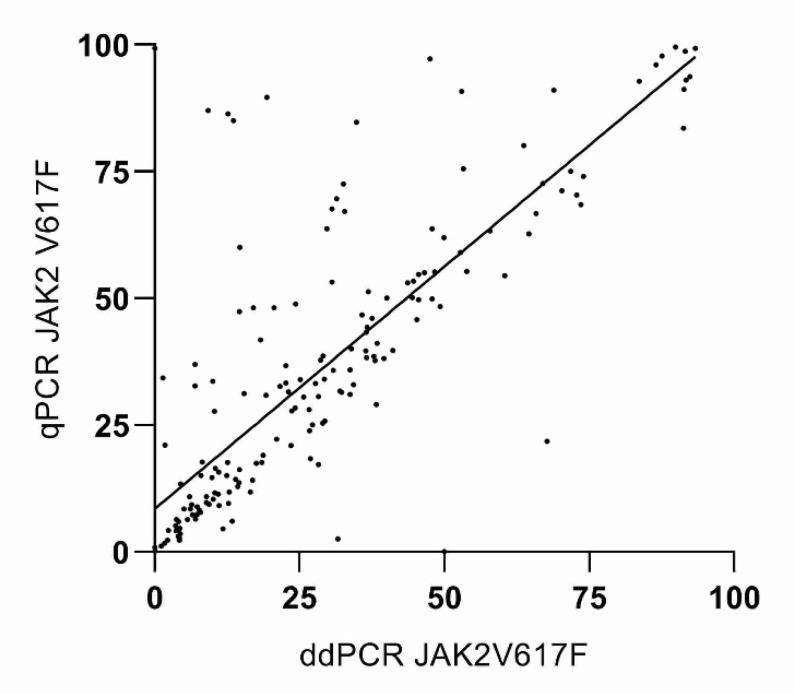
Correlation between qPCR and ddPCR.

The Spearman’s rho test, performed on patients’ samples, showed a moderate conformance between the two methods with a correlation coefficient of *r* = 0.83, *p* < 0.0001, range at 95% confidence interval 0.78 to 0.87 (Figure 2). 

**Figure 3:**
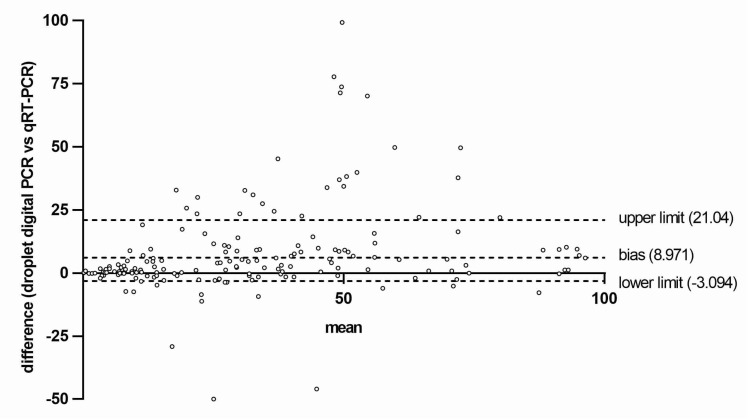
Bland–Altman analysis was used to plot differences between measurements by qPCR and ddPCR versus the mean of the sample. Dotted lines indicate bias (mean difference) and 95% of agreement (upper and lower limit +/− 2SD) between the two given methods.

Moreover, the bias between values resulting from qPCR versus ddPCR determinations was evaluated by using a Bland–Altman test. The bias was at the level of 8.9, and 95% of measurements of the limit of agreement lay from 3.09 to 21.04 (Figure 3). 

**Figure 4:**
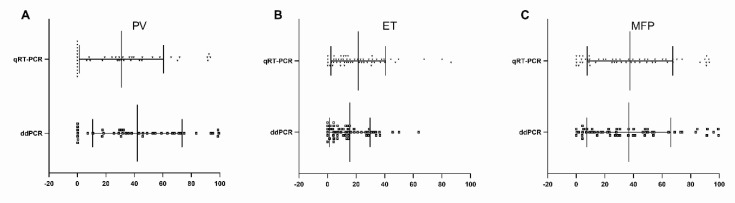
Comparison of median *JAK2 V617F* allele burden measured by qPCR and ddPCR. The Mann–Whitney test shows significant differences in PV patients (**A**), while no differences are observed among ET (**B**) and PMF patients (**C**).

Furthermore, the median of the JAK *V617F* mutation burden values among PV, ET, and PMF with the two methods was evaluated (Figure 4). 

Interestingly, statistically significant differences were observed among some of qPCR and ddPCR determinations. The *JAK2 V617F* allele burden values measured by using qPCR and ddPCR were significantly different (*p* = 0.033) among the 41 PV patients’ samples (Figure 4A). Instead, the values medians resulting from the two methods were comparable among ET and MPF patients’ samples (Figure 4B,C).

LoB values were 0.086% and 0.0034% for qPCR and ddPCR, respectively. Considering the assay performance, LoD was 0.66% for qPCR (95% confidence level) and 0.0092 (99% confidence level) for ddPCR. 

## 4. Discussion

Clinical response evaluation criteria in MPNs commonly include blood count, relief of anaemia by transfusion, reduction of splenomegaly, and symptoms control. However, the “ELN consensus criteria in ET and PV” also comprise molecular response [17]. The definition of molecular response is based on quantitative assessment of the allele burden of the *JAK2 V617F* specific molecular variant and, consequently, on its detection levels. A baseline value of mutant allele burden greater than 5% normally corresponds to a partial molecular response. However, the detection of a *JAK2 V617F* mutation at low levels is often too complex to be interpreted in clinical routine, and such determination is inevitably affected by the methodology used for molecular variant detection and quantification. Although the *JAK2 V617F* mutation is considered to be the major cause of MPN, it has been recently shown that *JAK2 V617F* positive neoplasms often develop in a background characterized by clonal hematopoiesis and other genetic alterations. Recently, different exome-analyses studies have identified the occurrence of age-related clonal hematopoiesis in healthy populations, to which the *JAK2 V617F* prevalence in the Danish population, close to 3%, could be ascribed [18]. By using highly sensitive methods, like ddPCR, we did not find a *JAK2 V617F* mutation in the control population, probably due to the low number of control samples used in our study. An increasing number of techniques have been developed for *JAK2 V617F* mutation identification by using molecular approaches including qPCR, such as high-resolution melt (HRM) [19], allele-specific oligonucleotide (ASO–PCR) [20], DNA direct sequencing [21], and recently ddPCR [22]. These techniques have different levels of LoD and specificity. Direct sequencing has a reported LoD of approximately 15% mutant alleles [21], ASO–RT–PCR has a LoD of around 1% [22], while ASO–ddPCR and locked nucleic acid (LNA) RT–PCR have greater sensitivity for detecting rare mutations, with a limit of detection of about 0.1% [23,24,25]. Several studies have suggested that the *JAK2 V617F* mutational load correlates with disease course and could therefore be a predictive marker. It has indeed reported that hematologic improvement correlates with a reduction in the *JAK2 V617F* mutational load [6,10,26]. Moreover, it has been demonstrated that measurement of the *JAK2 V617F* allele burden is able to predict relapse and outcome after allogenic stem cell transplantation (ASCT), thus representing a potential clinical tool for monitoring patients following ASCT [27]. For these reasons, a precise and sensitive method for quantification of the *JAK2 V617F* mutation is a compelling clinical need. In this study, we compared the technical performance profiles of qPCR and ddPCR for the evaluation of the *JAK2 V617F* mutation allele burden in a cohort of 195 *JAK2* positive patients with MPNs. In addition, our determinations were also compared with an international standard panel calibrator. Based on our results, the ddPCR method showed an optimal degree of accuracy and reproducibility, also providing a higher sensitivity in *JAK2 V617F* allelic burden quantification, if compared to qPCR. Notably, determinations obtained by using qPCR strongly depend on standards used for quantitation of target molecules. In contrast, ddPCR allows assessment of the total copy number of target molecules without the need for standard curves, thus facilitating standardization of quantitation. Despite ddPCR being an error-prone method that needs to be standardized, it is less sensitive to PCR inhibitors and therefore more robust than qPCR [28,29]. In addition, ddPCR proved to be an appropriate technique for the detection of mutations present at low allele frequencies because partitioning of the sample reduces wild-type background signals and therefore enhances accuracy of detection. In this study, we performed analysis of DNA reference samples containing very low levels of *JAK2 V617F* mutation burden. The advantage of this empirical approach is that objective data are used to compare the analytical responses of blank and low-concentration samples to determine conclusively what concentration of analyte is necessary to distinguish its presence from its absence and clearly distinguish it from a negative or blank sample [30]. However, in practice, both false positive and false negative signals can be produced from ddPCR assays. The threshold setting will determine false positive and false negative rates. A small proportion of false positives will have a large impact on the accuracy of assays with low numbers of target molecules, while a small proportion of false negatives will have a large impact on the accuracy of assays containing high numbers of target molecules. LoD and LoB are two important performance parameters to be established for validation of ddPCR measurements [31,32]. All data generated in this work have been analyzed by applying the same background noise level for all samples. The general approach to determine LoB involves determination of the distribution of values from multiple measurements of blank samples. LoD determination involves, instead, a series of measurements of samples containing very low levels of analyte. The 95th percentile of distribution of the blank signals typically determines the LoB. The LoD will be greater than the LoB [16]. Applied to ddPCR, LoD could be defined as the smallest number of target nucleic acid molecules that is statistically distinguishable from the background or negative control. One approach for estimating LoD and LoB for a ddPCR assay has been experimentally demonstrated in a study to determine lower limits of detection for cancer-related mutations [33,34]. As is well-known, accurate quantification of nucleic acid molecules by ddPCR is based on the correct sorting of single droplets into negative and positive populations, since the proportion of positive partitions is an integral component of the equation used to determine copy number concentration. Consequently, a key objective in optimization of a ddPCR assay is to maximize fluorescence amplitude difference between negative and positive partitions, and to minimize the number of partitions with intermediate fluorescence intensity. Practically, this can be achieved by optimizing the efficiency of the ddPCR assay.

Moreover, instrument-related factors can introduce bias into results even when assay protocol is followed meticulously. For example, accurate and reproducible temperature parameters during amplification are critical for reproducible results. Inaccurate thermal cycling temperatures, due to poor uniformity of temperature across the 96 wells of a thermocycler heating block, can result in no PCR amplification or low amplification efficiency in wells where optimal temperatures are not reached. To partially limit this inconvenience, we performed qPCR determinations with the same thermocycler for the all samples analyzed. In some cases, repeated-measures analyses performed with qPCR and ddPCR on the same sample produced discordant results (Figure 2 and Figure 3). This discrepancy could be due to the fact that the polymerase is less affected by PCR inhibitors and by other preanalytical factors in ddPCR when compared to qPCR.

By comparing results obtained from patients’ sample analysis, we found only a fair rate of concordance (Spearman *r* = 0.83) between the qPCR and ddPCR. Moreover, bias between the two methods was not close to the desirable limit of agreement for some of the measured points (Figure 3), indicating that ddPCR has precision and robustness greater than qPCR, especially in the measurement of low-allele *JAK2 V617F* frequencies.

## Figures and Tables

**Table 1:** Clinical characteristics of myeloproliferative neoplasms (MPNs) patients. PV—polycythemia vera, ET—essential thrombocytemia, PMF—primary myelofibrosis.

Characteristics	ET	PV	PMF	Control
Number of patients	88	63	44	50
Median age, years (range)	66 (32–96)	68 (37–89)	67 (38–94)	58 (19–66)
Gender, M/F	49 F 39 M	21 F 42 M	18 F 26 M	8 F 12 M
Median hemoglobin, g/dl (range)	13.42 (8.1–17)	16.1 (10.3–19.8)	12.42 (7.8–23.2)	14.9 (12.2–16.8)
Median hematocrit, % (range)	41.65 (24.4–52)	50.4 (33.7–63)	38.68 (22.6–73.9)	43.6 (26–61.2)
Median red blood cell, M/µL (range)	5.02 (3.29–6.1)	6.23 (3.39–8.96)	6.16 (2.88–50.05)	5.3 (4.19–5.3)
Median white blood cell, K/µL (range)	8.1238 (2.9–14.6)	10.46 (4.82–46.96)	16.22 (1.46–70.91)	7.87 (4.9–11.6)
Median platelets, K/µL (range)	651.73 (93–1352)	434.08 (108–879)	387.03 (15–1492)	490.3 (340–790)
